# Characterization of the initial degradation mechanism involved in *C*-glycosylated isoflavone puerarin in soil microorganism

**DOI:** 10.1128/aem.01593-25

**Published:** 2025-11-20

**Authors:** Takuto Kumano, Satomi Watanabe, Masakazu Kusakari, Yuzu Terashita, Yoshiteru Hashimoto, Michihiko Kobayashi

**Affiliations:** 1 Graduate School of Life and Environmental Sciences, University of Tsukuba 13121, Tsukuba, Ibaraki, Japan; 2 Microbiology Research Center for Sustainability (MiCS), University of Tsukuba 13121, Tsukuba, Ibaraki, Japan; 3 Tsukuba Institute for Advanced Research (TIAR), University of Tsukuba 13121, Tsukuba, Ibaraki, Japan; 4 Center for Quantum and Information Life Science (QILS), University of Tsukuba 13121, Tsukuba, Ibaraki, Japan; Washington University in St Louis, St. Louis, Missouri, USA

**Keywords:** herbal medicine, C-C bond cleavage, metabolism, flavonoid

## Abstract

Puerarin is C8-glycosylated daidzein and an active ingredient in the Chinese herbal medicine “Kakkonto.” It is used in the early stage of cold treatment. A previous study reported that intestinal bacteria can remove the sugar moiety from puerarin. Despite their potential roles, *C*-glycosylated flavonoid-metabolizing microorganisms, including those acting on puerarin, have only been found in the gut but not been identified in natural environments. In this study, we identified a puerarin-catabolizing microorganism by screening from soil and clarified two-step deglycosylation reaction. The first step in the metabolism of puerarin was the oxidation of a sugar moiety, catalyzed by a FAD-dependent oxidase named PurA. PurA is the first example of a *C*-glycoside oxidase that showed activity toward isoflavone-*C*-glycosides. Notably, PurA exhibited a broader substrate specificity, acting on both C6- and C8-glycosylated flavonoids. This feature was not found in previously reported *C*-glycoside oxidases and could be useful to synthesize aglycone compounds from *C*-glycosides in the future. The second step involved the cleavage of the C-C bond between the sugar moiety and daidzein, which was catalyzed by a *C*-glycoside deglycosylation enzyme named PurBC. Surprisingly, the initial enzyme in puerarin catabolic pathway in this strain differs from that found in the intestinal bacteria. These findings indicate that, in natural environments, bacteria distinct from intestinal microbiota are responsible for puerarin degradation. Our findings provide a novel insight into microbial *C*-glycoside metabolism in soil environments.

**IMPORTANCE:**

Plants, fungi, bacteria, and insects synthesize various kinds of unique compounds. Those synthesized compounds are generally degraded by microorganisms; otherwise, they would accumulate on the surface of the Earth. However, in many cases, their fate in the natural environment remains poorly understood. In this study, we isolated a bacterium from soil by the enrichment culture method and identified it as *Paenarthrobacter* sp. No. 37. This strain was able to grow in the medium containing puerarin as the sole carbon source. It revealed that strain No. 37 can utilize puerarin as a source of carbon and energy. Furthermore, a puerarin degradation activity was induced upon the addition of puerarin to the culture medium. Thus, we successfully identified a bacterium that physiologically catabolizes puerarin and potentially other *C*-glycosylated compounds. By uncovering *C*-glycoside metabolism in natural environments, our findings shed light on the part of microbial contribution to the degradation and regeneration of plant-derived natural compounds in biogeochemical cycles.

## INTRODUCTION

Plants are known to synthesize a wide variety of secondary metabolites, many of which are stored in glycosylated forms. The glycosylated compounds are more stable and water-soluble than the non-glycosylated original compounds (1). Depending on the type of glycosidic linkage, glycosides are classified as *O*-, *C*-, *S*-, and *N*-glycosides. Among them, *C*-glycosides have different features from the others because the anomeric carbon of the sugar moiety is directly bonded to the carbon skeleton of an aglycon, which is the non-sugar moiety of a glycoside. *C*-Glycosides are rare compared with the most abundant glycoside, *O*-glycosides, but more than a hundred types have been isolated from natural sources (2). For example, puerarin is included in the root of the kuzu plant (*Pueraria lobata* subsp. *lobata*), and the powder made from the root is used in traditional Japanese foods, such as kuzu-mochi, kuzu-yu, and is also known as the Chinese herbal medicine “Kakkonto.” Recent studies have shown that puerarin has useful physiological activities in the treatment of cancer and diabetes (3, 4).

*C*-Glycosides taken orally from foods or herbal medicines were reported to be metabolized by “intestinal” bacteria (5–8), and the puerarin-metabolizing enzymes were identified as DgpA and DgpBC (9). DgpA is an NAD-dependent oxidoreductase and catalyzes dehydration of the C3-OH of the sugar moiety, and DgpBC catalyzes the C-C bond cleavage between the sugar moiety and the aglycone.

While the metabolic pathway of puerarin by intestinal bacteria has been reported, the microorganisms, metabolic pathways, and enzymes responsible for puerarin metabolism in soil environments remain unknown. In this study, we isolated a puerarin-catabolizing microorganism from screening of soil bacteria and identified each of two enzymes that catalyze the two-step deglycosylation reaction involved in puerarin metabolism. These findings provide new insights into the *C*-glycoside metabolism in natural environments.

## MATERIALS AND METHODS

### Screening of puerarin-catabolizing microorganisms

Using culture media containing puerarin as the sole carbon source, puerarin-metabolizing microorganisms were isolated from soil collected from the University of Tsukuba by the following enrichment method. Step 1: The collected soil was added to 10 mL of culture media consisting of 0.025% (w/v) puerarin (dissolved in methanol), 1% (w/v) (NH_4_)_2_SO_4_, 0.05% (w/v) KH_2_PO_4_, 0.05% (w/v) K_2_HPO_4_, 0.05% (w/v) MgSO_4_·7H_2_O, 0.0005% (w/v) FeSO_4_·7H_2_O, and 20% (v/v) tap water (adjusted to pH 7.0 with NaOH), followed by incubation at 28°C for 1 week. Step 2: 2% (v/v) of the cultivated medium was added to the fresh medium, followed by incubation at 28°C for 1 week. Step 2 was repeated twice.

The culture broth was diluted 10^4^-fold and plated on agar medium and incubated at 28°C. Colonies were picked up and isolated. The isolated colonies were cultured and mixed with an equal volume of autoclaved 40% (w/v) glycerol and stored at −80°C (Table S1).

### Culture method using nutrient medium

Each of the isolated microorganisms was cultured at 28°C with shaking in the 2×YT + puerarin medium, which consisted of 1% (w/v) yeast extract (DIFCO, NJ, USA), 1.6% (w/v) Tryptone (DIFCO, NJ, USA), 0.5% (w/v) NaCl (Nacalai, Kyoto, Japan), and 0.005% (w/v) puerarin (Sigma Aldrich, MO, USA). After 2 days of pre-culturing in 5 mL of medium, the main culture was carried out by transferring 2% (v/v) of the pre-culture broth to 1 L of the medium.

### Measurement of puerarin degradation activity and identification of the metabolites of strain No. 37

Puerarin-metabolizing microorganism No. 37 was cultured in the 2×YT + puerarin medium. The cells were harvested by centrifugation (4,000 × *g*, 10 min, 4°C), and they were resuspended in 1 mL of 100 mM Tris-HCl (pH 8.0). The cells were then disrupted by sonication (150 W, 20 min, INSONATOR 201M, KUBOTA, Tokyo, Japan), and the cell debris was removed by centrifugation (27,000 × *g*, 10 min, 4°C) to prepare cell-free extracts. Two hundred microliters of the reaction mixture contained 20 µL of 1 M Tris-HCl (pH 8.0), 2 µL of 100 mM puerarin (in methanol), 10 µL of the cell-free extracts and distilled water. After incubation at 28°C for 90 min, the reaction was stopped by adding 200 µL of methanol. Reaction samples were analyzed by high performance liquid chromatography (HPLC, prominence 20A, Shimadzu, Kyoto, Japan) and liquid chromatography-mass spectrometry (LC/MS, LCMS-8040, Shimadzu, Kyoto, Japan).

### Extraction of chromosomal DNA and determination of draft genome sequence

Total DNA from strain No. 37 was prepared as follows: the strain was cultured at 28°C for 2 days in 100 mL of 2×YT medium (0.1% [w/v] yeast extract, 0.16% [w/v] Tryptone, and 0.05% [w/v] NaCl). Cells were harvested by centrifugation and suspended in 2 mL of 100 mM potassium phosphate buffer (KPB) (pH 7.0). Cells were disrupted using the Shake Master Auto (BMS, Tokyo, Japan), and cell-free extracts were prepared. DNA was purified by extracting the lysate with phenol/chloroform/isoamyl alcohol (25/24/1; v/v/v), followed by precipitation with isopropanol, treatment with RNase (Takara, Shiga, Japan), and then reprecipitation with ethanol. The draft genome sequencing of strain No. 37 was performed using an Illumina Hiseq platform (Illumina, CA, USA). We obtained 47.4 million reads of a 100 bp paired-end reads. A total of 114 contigs comprising 195–412,147 bp were assembled. The genes in the draft genome sequence were annotated using DFAST (10) (https://dfast.ddbj.nig.ac.jp).

### Purification and identification of PurBC

Strain No. 37 was cultured in 1 L of the 2×YT + puerarin medium. The culture broth was centrifuged at 5,000 rpm for 10 min at 4°C, and the cells were collected. The cells were suspended in 80 mL of 100 mM Tris-HCl (pH 8.0) and disrupted by sonication (150 W, 20 min, INSONATOR 201M, KUBOTA, Tokyo, Japan). The disrupted solution was centrifuged at 15,000 rpm at 4°C for 10 min to prepare cell-free extracts, and the cell debris was removed by centrifugation (27,000 × *g*, 20 min, 4°C) to prepare cell-free extracts. Purification of the puerarin-metabolizing enzyme (PurBC) was carried out through the steps described below. Each column chromatography was performed on an AKTA purifier (GE Healthcare, Buckinghamshire, UK).

#### Step 1: Ammonium sulfate fractionation

The cell-free extracts were fractionated with ammonium sulfate (40% saturation). The supernatant was applied to the column chromatography after filtration.

#### Step 2: TOYOPEARL Butyl-650M

The resulting supernatant was applied onto a TOYOPEARL Butyl-650M (TOSOH) column equilibrated with 20 mM Tris-HCl buffer (pH 8.0) containing 1 M ammonium sulfate. The enzyme was eluted by decreasing the concentration of ammonium sulfate (1 M to 0 M).

After dialysis of each fraction with 20 mM Tris-HCl (pH 8.0), puerarin degradation activity was measured.

#### Step 3: ResourceQ

The active fractions were placed on a ResourceQ column (GE Healthcare) equilibrated with 20 mM Tris-HCl buffer (pH 8.0). The enzyme was eluted by increasing the concentration of NaCl (0 to 1 M). Puerarin degradation activity was measured.

#### Step 4: BioassistQ

The active fractions were dialyzed against 20 mM Tris-HCl buffer (pH 8.0), and then placed on a BioassistQ column (GE Healthcare) equilibrated with 20 mM Tris-HCl buffer (pH 8.0). The enzyme was eluted by increasing the concentration of NaCl (0 to 1 M). Puerarin degradation activity was measured.

#### Step 5: Superose12 10/300GL

The active fractions were concentrated by using Amicon Ultra-0.5 mL (Millipore) to reduce the volume, and then placed on a Superose12 10/300GL column (GE Healthcare). The conditions for the elution were as follows: flow rate, 1 mL/min; and buffer, 20 mM Tris-HCl (pH 8.0) containing 0.1 M NaCl.

#### Step 6: Transfer the target enzyme to PVDF membrane and determine the *N*-terminal amino acid sequence

The fraction samples containing the target enzyme obtained by purification were subjected to SDS-PAGE. The transfer was performed at a voltage of 15 V for 60 min. After the transfer was completed, the PVDF membrane was stained. The target protein band was cut out, and the *N*-terminal amino acid sequence was determined.

### Cloning and heterologous expression of purA and purBC

*purA* and *purBC* were amplified using “*PurA_fw* and *PurA_rv*” and “*PurC-fw* and *PurB_rv*” primer sets, respectively (Table S2). The PCR product was cloned into the linearized pET24a(+) vector by using In-fusion (Clontech Laboratories Inc., CA, USA). The resulting plasmid was designated as pET24a(+)-*purA* and pET24a(+)-*purBC. Escherichia coli* Rosetta2 (DE3) cells harboring the plasmid pET24a(+)-*purA* or *purBC* were cultivated in 1 L of liquid 2×YT medium containing 50 µg/mL kanamycin and 30 µg/mL chloramphenicol, and grown at 37°C to an OD_600_ of 1.5. The temperature was lowered to 18°C, and isopropyl-β-D-thiogalactoside (IPTG) was added to a final concentration of 0.5 mM. The cells were cultured for a further 20 h and then harvested. Twenty milliliters of 20 mM Tris-HCl buffer (pH 8.0) was added to the pellet (20 g). The cells were disrupted with the sonicator described above. The lysate was centrifuged at 27,000 × *g* at 4°C for 20 min. Each of the recombinant PurA and PurBC was purified by using a His Trap HP column (GE Healthcare, IL, USA).

### Enzyme assay for PurA

Measurement of enzyme activity was performed as follows. One hundred microliters of the reaction mixture [5 µL of 0.015 mg/mL PurA, 5 µL of 1 M Tris-HCl (pH 8.0), 0.1 mM FAD, and 1 µL of 10 mM puerarin (in milliQ water)] was used. One unit of puerarin-metabolizing activity was defined as the amount of enzyme required to catalyze the formation of 1 µmol of the reaction product per min. Specific activity is expressed as units per milligram of protein.

The reaction was initiated by adding PurA, followed by incubation at 28°C for an appropriate time. After incubation, the reaction was stopped by adding 100 µL of acetonitrile.

### Determination of kinetic parameters

Two hundred microliters of the reaction mixture consisted of 20 µL of 0.015 mg/mL PurA, 4 µL of 1 M Tris-HCl (pH 8.0), 1 µL of 50 mM *N*-ethyl-*N*-(2-hydroxy-3-sulfopropyl)−3,5-dimethoxyaniline sodium salt (DAOS), 4 µL of 50 mM 4-aminoantipyrine, 4 µL of 5000 U/mL peroxidase, and from 0.025 mM to 0.1 mM puerarin. The reactions were initiated on the addition of PurA, followed by incubation at 28°C. The produced H_2_O_2_ couples 4-aminoantipyrine and DAOS to yield a blue dye, which can be detected at 595 nm by spectrophotometry (SpectraMax 190, Shimadzu, Kyoto, Japan). The experiments were carried out in triplicate independently. *k*_cat_ values were calculated using a molecular mass of 55,563 for PurA.

### Temperature dependency

For the estimation of thermal dependency of PurA, 200 µL of the reaction mixture consisted of 177 µL of milliQ water, 10 µL of 0.015 mg/mL PurA, and 10 µL of 1 M Tris-HCl (pH 8.0), and 1 µL of 100 mM FAD and 2 µL of 100 mM puerarin were used. The experiments were carried out at 10°C, 20°C, 25°C, 30°C, 35°C, 40°C, 45°C, 50°C, 60°C, and 80°C for 15 min. A Chill Heat CHT-101 (IWAKI Asahi Techno Glass, Tokyo, Japan) was used for incubation at 10°C to 50°C, and a Dry Thermo Unit DTU-1B (TAITEC, Tokyo, Japan) was used for incubation at 60°C and 70°C. The amounts of reaction products were determined by HPLC.

For the estimation of thermal dependency of PurBC, a pre-reaction mixture_A containing 74 µL of milliQ water, 5 µL of 10 mM FAD, 5 µL of 0.015 mg/mL PurA, 5 µL of 1 M Tris-HCl (pH 8.0), and 1 µL of 100 mM puerarin (in methanol) was incubated at 28°C for 2 h to convert puerarin to 3″-oxo-puerarin. Then, 5 µL of 0.027 mg m^−1^ PurBC was added to the pre-reaction mixture_A. The reactions were performed at 20°C, 25°C, 30°C, 35°C, 40°C, 45°C, 50°C, 60°C, and 70°C for 15 min and stopped by adding 100 µL of acetonitrile. The Chill Heat CHT-101 (IWAKI Asahi Techno Glass, Tokyo, Japan) was used for the incubations from 10°C to 50°C, and a Dry Thermo Unit DTU-1B (TAITEC, Tokyo, Japan) was used for the incubations at 60°C and 70°C. The amounts of reaction products were determined by HPLC. The experiments were repeated three times independently.

### pH dependency

The pH dependence of PurA was examined at pH 4.2, 5.2, 6.4, 7.4, 8.3, 8.7, 9.0, 10.0, 11.2, and 12.2. Two hundred microliters of the reaction mixture consisted of 177 µL of milliQ water, 10 µL of 0.0015 mg/mL PurA, 10 µL of 0.4 M Britton-Robinson buffer, and 2 µL of 100 mM puerarin. The experiments were carried out for 15 min at 28°C. The reaction was stopped by adding 200 µL of acetonitrile. The amount of the reaction product was determined by HPLC.

For the estimation of pH dependency of PurBC, a pre-reaction mixture_A containing 49 µL of milliQ water, 5 µL of 10 mM FAD, 5 µL of 0.015 mg/mL PurA, 5 µL of 0.1 M Tris-HCl (pH 8.0), and 1 µL of 100 mM puerarin (in methanol) was incubated at 28°C for 2 h to convert puerarin to 3″-oxo-puerarin. Then, 5 µL of 0.027 mg mL^−1^ PurBC and 25 µL of 0.4 M Britton-Robinson buffer (pH 5.5–9.5 [0.5 pH units]) were added to the pre-reaction mixture_A. The reactions were performed for 15 min at 28°C and stopped by adding 100 µL of acetonitrile. The amounts of reaction products were determined by HPLC. The experiments were repeated three times independently.

### Inhibitors and metals

For the determination of the effects on PurA, 100 µL of reaction mixtures containing 84 µL of milliQ water, 5 µL of 10 mM FAD, 5 µL of 0.015 mg/mL PurA, 5 µL of 1 M Tris-HCl (pH 8.0), 1 µL of 100 mM puerarin (in methanol), and 1 mM of each inhibitor (see below) was incubated at 28°C for 15 min. The experiments were carried out in triplicate. The amounts of reaction products were determined by HPLC.

For the determination of the effects on PurBC, a pre-reaction mixture_B containing 79 µL of milliQ water, 5 µL of 10 mM FAD, 5 µL of 0.015 mg/mL PurA, 5 µL of 1 M Tris-HCl (pH 8.0), and 1 µL of 100 mM puerarin (in methanol) was incubated at 28°C for 2 h to convert puerarin to 3″-oxo-puerarin. Then, 1 µL of 100 mM inhibitors (see below) and 5 µL of 0.027 mg/mL PurB/C were added to the pre-reaction mixture_B and incubated at 28°C for 30 min. The experiments were carried out in triplicate. The amounts of reaction products were determined by HPLC.

The reagents listed below were used as inhibitors or metals (those not specifically mentioned were dissolved in MilliQ to 100 mM). Metals; LiCl, PbCl_2_ (in methanol), NaCl, HgCl_2_, MgCl_2_•6H_2_O, NiCl_2_•6H_2_O, CaCl_2_•2H_2_O, CuCl_2_•2H_2_O, BaCl_2_•2H_2_O, FeSO_4_•7H_2_O, MnCl_2_•4H_2_O, FeCl_3_, ZnCl_2_, RbCl, CdCl_2_•2.5H_2_O, SrCl_2_•6H_2_O, CoCl_2_•6H_2_O, CsCl, AlCl_3_•6H_2_O, Na_2_MoO_4_•2H_2_O: Inhibitors; 5,5′-dithio-bis-2-nitrobenzoate (DTNB) (dissolved in 20 mM Tris-HCl (pH 8.0) to 2 mM); iodoacetate; *N*-ethylmaleimide (NEM) (dissolve by exposing to boiling water for 1 min); *p*-chloromercuribenzoate (PCMB) (dissolved in 1 M NaOH); hydroxylamine•HCl; phenylhydrazine•HCl; semicarbazide•HCl; aminoguanidine•H_2_CO_3_ (dissolve to 50 mM by exposing to boiling water for 1 min); α,α'-dipyridyl (dissolved in 100 µL methanol and diluted with 10 mL of 20 mM Tris-HCl (pH 8.0) to 4 mM); *o*-phenanthroline•H_2_O (dissolved in 100 µL methanol and diluted with 10 mL of 20 mM Tris-HCl (pH 8.0) to 4 mM); EDTA (dissolve to 10 mM); diethyldithiocarbamate•3H_2_O; NaN_3_; KCN; dithiothreitol (DTT); 2-mercaptoethanol; sodium hydrosulfate (Na_2_SO_4_); H_2_O_2_ (dissolve 10 µL of 30%H_2_O_2_ in 990 µL of milliQ); ammonium persulfate; diisopropyl fluorophosphate (DFP), (17.5 µL dissolved in 1 mL methanol).

### Determination of molecular mass by gel filtration chromatography

Gel-filtration chromatography was performed for the determination of each molecular mass of PurA and PurBC. PurA (0.76 mg/mL) and PurBC (1.37 mg/mL) were applied to a SuperdexTM 200 10/300GL (GE Healthcare). The analyses were carried out using an AKTA purifier (GE Healthcare). The conditions for the analysis were as follows: flow rate, 1 mL min^−1^; and buffer, 20 mM Tris-HCl (pH 8.0) containing 0.1 M NaCl. The standard proteins, i.e., glutamate dehydrogenase (290 kDa), lactate dehydrogenase (140 kDa), enolase (67 kDa), myokinase (32 kDa), and cytochrome *c* (12.4 kDa) (MW-Marker [HPLC], Oriental Yeast Co., Ltd., Ohtsu, Japan), were applied before and after sample injection. The molecular mass of the enzyme was calculated from the mobilities of the standard proteins.

### Metal dependency of PurBC

The purified recombinant PurBC was treated with 5 mM EDTA and 5 mM Tiron at 4°C with slow rotation for 3 days, and then the excess EDTA was removed by dialysis with 20 mM Tris-HCl (pH 8.0). After a pre-reaction mixture_C containing 69 µL of milliQ water, 5 µL of 0.1 mM FAD, 5 µL of 0.015 mg/mL PurA, 5 µL of 1 M Tris-HCl (pH 8.0), and 5 µL of 10 mM puerarin (in methanol) was incubated at 28°C for 1 h, 5 µL of 0.027 mg/mL PurBC was added to the pre-reaction mixture_C without or with 1 µL of 1 mM divalent metal ion (Mn^2+^, Ni^2+^, Mg^2+^, Fe^2+^, Cu^2+^, Co^2+^, Zn,^2+^ and Ca^2+^). All measurements were conducted in triplicate. The reactions were terminated with methanol, and the mixtures were centrifuged at 20,000 × *g* for 10 min for further HPLC analysis.

### Substrate specificity of PurA and PurBC

Each of the following compounds was examined for substrate specificity analysis at a final concentration of 0.1 mM: carminic acid, mangiferin, homoorientin, isovitexin, puerarin, orientin, aloesin, emodin-8-glucoside, genistin, rutin, naringin, apigetrin, daidzin, and aloenin. The reaction mixture for PurA was composed of 80 µL of milliQ water, 5 µL of 0.1 mM FAD, 5 µL of 0.015 mg/mL PurA, 5 µL of 1 M Tris-HCl (pH 8.0), and 5 µL of 10 mM each substrate. The reaction mixture for PurBC was composed of 75 µL of milliQ water, 5 µL of 0.1 mM FAD, 5 µL of 0.015 mg/mL PurA, 5 µL of 1 M Tris-HCl (pH 8.0), 5 µL of 10 mM each substrate, and 5 µL of 0.027 mg/mL PurBC. The reaction mixture was pre-incubated at 28°C for 1 h before adding PurBC. The reaction products were identified by mass spectrometry. Each reaction was performed in triplicate.

### Database search of PurABC and DgpABC homologs

The query was prepared by concatenating the amino acid sequences of PurA, PurB, and PurC in this order. A tblastn search (https://blast.ncbi.nlm.nih.gov/Blast.cgi) was then performed against the core nucleotide database. Five hundred sequences were obtained and filtered using an E-value ≤1e-50 and query coverage between 80% and 100%. The source organisms of the selected sequences were classified at the phylum level. The same procedure was applied to DgpABC.

## RESULTS

### Screening of puerarin-catabolizing microorganisms

**Fig 1:**
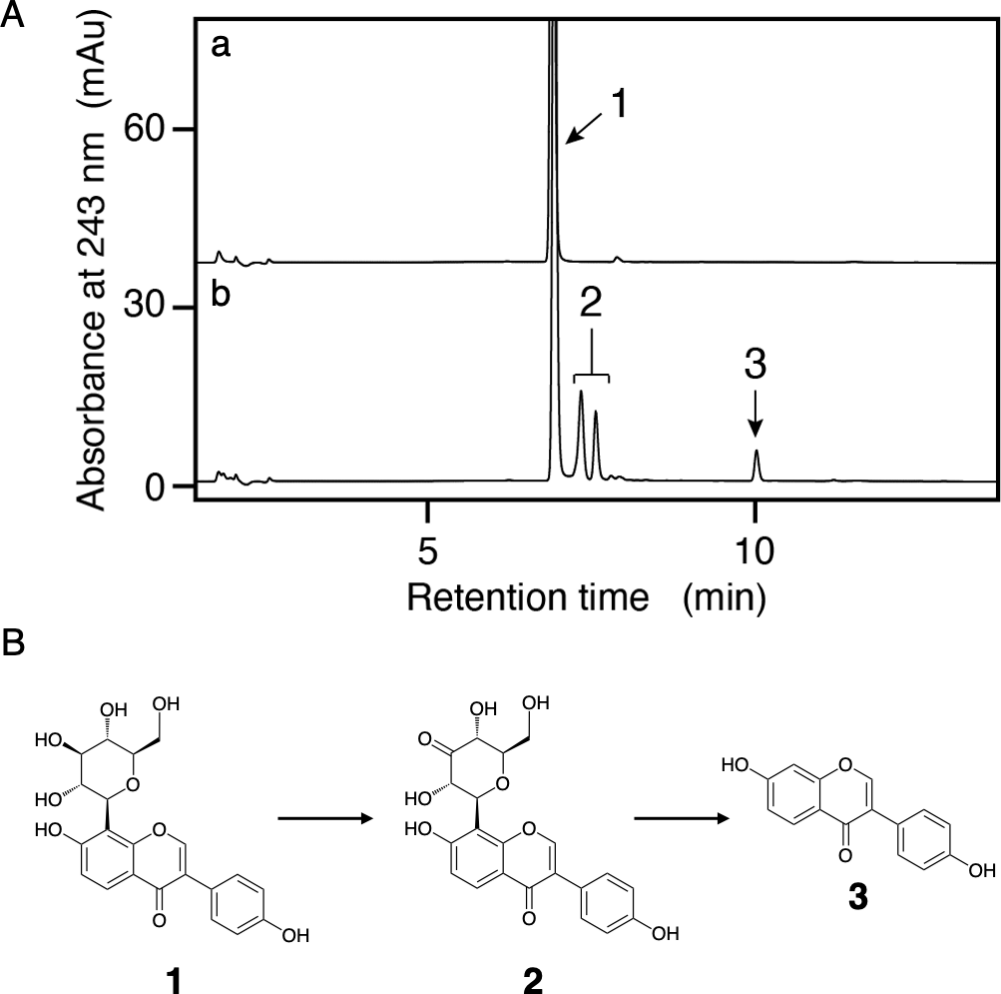
Analysis of the puerarin-metabolizing activity of the cell-free extracts of strain No. 37. (**A**) HPLC chromatograms. a, only puerarin was incubated. b, puerarin was incubated with the cell-free extracts prepared from No. 37 cells. The numerical values represented in this figure correspond to puerarin 1, 3″-keto-puerarine and its isomers 2, and daidzein 3. (**B**) Proposed reaction steps of deglycosylation involved in the puerarin metabolism in strain No. 37.

The soil samples used for microbial screening were collected from several locations around the University of Tsukuba. Using the enrichment culture method described in Materials and Methods, we isolated microorganisms capable of growing on a medium containing puerarin as the sole carbon source. We isolated 45 colonies from the puerarin sole carbon medium. Among them, strain No. 37 showed the most potent puerarin-degrading activity (Fig. 1A). Therefore, strain No. 37 was used for further experiments. In terms of the 16S rRNA gene sequence, strain No. 37 showed 99.3% identity with *Paenarthrobacter histidinolovorans* strain DSM 20115^T^.

Strain No. 37 demonstrated puerarin-degrading activity when cultivated on the 2×YT medium containing the substrate. Conversely, such activity was observed very weakly when the strain was grown on a medium lacking puerarin (Fig. S1). These results indicated that the puerarin-degrading activity was induced by puerarin.

### Structure determination of the reaction products converted from puerarin

The LC/MS results demonstrated that puerarin underwent conversion into three distinct products, designated as compounds P1, P2, and P3. Two of the products (P1 and P2) exhibited the same m/z 413 [M-H]^-^, indicated a mass reduction of two from the substrate. Based on our previous findings obtained from carminic acid metabolism (11), these products were suggested to be 3″-keto puerarin two and its isomer (Fig. 1). P3 displayed an m/z 253 [M-H]^-^, agreed with the mass and retention time of daidzein **3**, the aglycone of puerarin. These results indicated that the sugar moiety of puerarin was cleaved via a two-step reaction. The first reaction was a dehydrogenation of puerarin to form P1 and P2 in a reduction of the relative molecular mass by two. The second reaction was the cleavage of the sugar moiety of P1 and P2 to form daidzein (P3) (Fig. 1B).

### Purification of C-C bond cleavage enzyme (PurBC), cloning, and expression of the structural gene

We attempted to purify the enzyme catalyzing the second step of puerarin degradation from strain No. 37. We carried out the purification of the 3′-keto-puerarin-converting enzyme. Proteins in cell-free extracts of strain No. 37 were precipitated with ammonium sulfate, and the target enzyme was further purified by using column chromatographies, as described in Materials and Methods (Table S3). The yield of the target enzyme was 1.3%, and the overall increase in the specific activity was 19-fold. After purification steps, the target enzyme was observed as approximately 40 and 16 kDa protein bands on SDS-PAGE (Fig. S2). We named 40 kDa protein as PruB and 16 kDa protein as PurC. *N*-Terminal partial amino acid sequences of PurB and PurC were determined to be TASNNYSLPTIGSGLTLYSF and SATERV, respectively. From the draft genome sequence data for No. 37, we identified corresponding open reading frames (ORF) of PurB and PurC (see the supplemental results). The deduced amino acid sequences indicated that PurB and PurC consisted of 344 and 136 amino acids with a theoretical molecular mass of 38.3 and 15.0 kDa, respectively. These values were in good agreement with the protein sizes observed on SDS-PAGE.

We cloned the 1442 bp region, including *purB* and *purC*, and constructed a plasmid pET24a(+)_*purBC*. A His-tag was added to the *C*-terminal of *purB*. PurB and PurC were co-expressed in *E. coli* BL21 Star (DE3) harboring pET24a(+)_*purBC*, and the His-tagged PurB was purified together with PurC using the Ni-affinity column (Fig. S3).

### Identification and heterologous expression, kinetic parameters of *C*-glycoside oxidase (PurA)

From the draft genome sequence data for strain No. 37, an open reading frame (ORF) of 1,551 nucleotides was identified at the downstream region of *purBC* (Fig. S4 and supplemental results). The amino acid sequence of the ORF exhibited similarity to CarA, which is a *C*-glycoside oxidase involved in carminic acid metabolism. The 1.5 kb region of the PurA-coding gene was inserted into an expression vector, pET24a(+) (Tables S1 and S2). The recombinant PurA was expressed as a His-tagged protein in *E. coli* Rosetta2 (DE3) and purified by the Ni-NTA column chromatography (Fig. S3). Apparent steady-state kinetic constants of the purified recombinant PurA were estimated for puerarin by quantitative measurement. Non-linear curve fitting to a model showed that *K*_m_ was 0.0434 ± 0.0066 mM, and *V*_max_ was 4.05 ± 0.14 (µmol/min/mg) and *k*_cat_ of 3.75 ± 0.13 s^−1^. Further experiments were conducted utilizing the recombinant PurA.

### Size-exclusion chromatography of PurA and PurBC

A peak of PurA appeared at 14.4 min (Fig. S5), and the molecular mass of PurA was determined to be about 47.8 kDa by substituting it into the calibration curve; the estimated molecular mass of PurA based on its amino acid sequence is 55,563 Da, suggesting that PurA is a monomer. After PurB and PurC were co-expressed in *E. coli* and purified by the Ni-affinity column, we carried out gel-filtration chromatography. PurB and PurC were excluded in the same fraction (Fig. S6). The molecular size was estimated to be 52.4 kDa by using the standard protein marker. The calculated mass of PurB and PurC was 38.3 and 15.0 kDa, respectively. This finding indicated that PurB and PurC formed a heterodimer.

### Temperature and pH dependency of PurA and PurBC

We examined the effects of temperature and pH on PurA and PurBC activity. PurA was incubated with puerarin at various temperatures and in various pH values. PurA showed the highest activity at 30°C, 80% activity at 40°C, 20% activity at 50°C, and a complete loss of activity at 60°C (Fig. S7A). In the pH range of 7.5–8.3, PurA demonstrated 100% activity, while activity levels were less than 40% at pH values below 6.5 and above 9.0 (Fig. S7B). On the other hand, PurBC showed the highest activity at 40°C, 45% activity at 40°C, and no activity over 60°C (Fig. S7C). In the pH range of 7.5–8.3, PurBC demonstrated nearly 100% activity, while at pH 7.0 and pH 9.0, its activity was recorded at 60% and 40%, respectively. PurBC exhibited no activity at pH 6 and pH 10 (Fig. S7D).

### Metal dependency of PurBC

The specific activity of PurBC, which was not treated by chelator, was shown as 100% in Fig. S8. The metal-free PurBC was prepared by dialysis for 3 days with chelating agents EDTA and tiron. Despite this prolonged dialysis period, 10% of the activity remained. The activity of the treated PurBC was restored to 100% on the addition of Mg^2+^, Mn^2+^, and Co^2+^ and partially recovered by Ni^2+^ and Ca^2+^ (Fig. S8). The activity of the enzyme remained inactive in the presence of Cu^2+^ and Zn^2+^.

### The effects of various compounds on the enzyme activities of PurA and PurB

The activity of PurA was inhibited by more than 95% by PbCl_2_, FeSO_4_, FeCl_3_, ZnCl_2_, MnCl,_2_ and 10%–60% by CoCl_2_, CuCl_2_, HgCl_2_, CdCl_2_, and AlCl_3_ (Table S4). In non-metal inhibitors, the activity of PurA was inhibited by more than 40% by diisopropyl fluorophosphate, which is an inhibitor of serine protease (Table S5). On the other hand, the activity of PurBC was inhibited by more than 98% by ZnCl_2_, HgCl_2_, CuCl_2_, PbCl,_2_ and 40%–70% by NiCl_2_, FeSO_4_, and CdCl_2_ (Table S4). In non-metal inhibitors, the activity of PurBC was inhibited by more than 50%–90% by a SH-inhibitor PCMB, chelating agents *o*-phenanthroline•H_2_O, diethyldithiocarbamate•3H_2_O, KCN, α,α′-dipyridyl, and a reducing agent DTT (Table S5).

### Substrate specificity of PurA and PurBC

**Fig 2:**
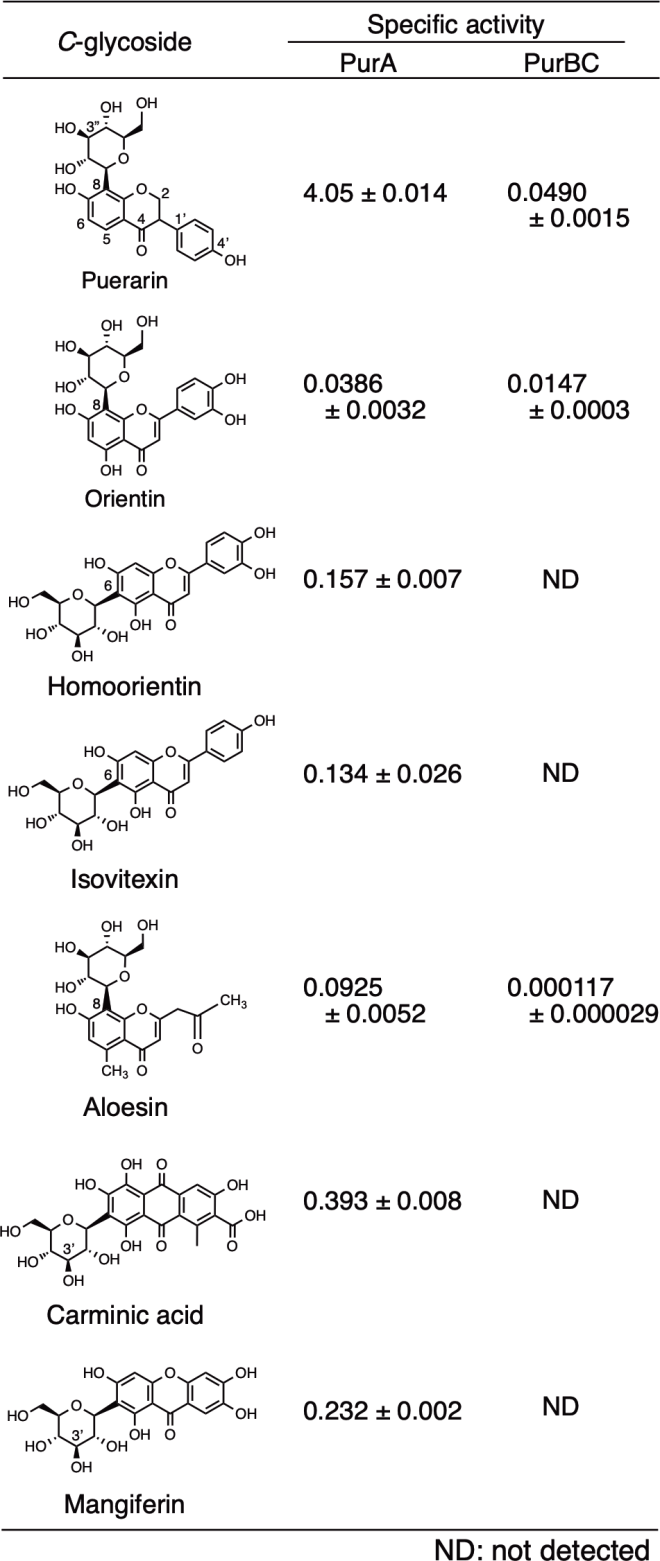
Substrate specificities of PurA toward *C*-glycosides.

We investigated the substrate specificities of PurA and PurBC toward *C*- and *O*-glycosides. When *C*-glycosides were used as substrates for PurA, all of them were oxidized, and the corresponding 3-keto-*C*-glycosides were formed. PurA exhibited the 1/10 activity toward carminic acid and mangiferin, the 1/20 activity toward C6-glycosylated flavones (homoorientin and isovitexin), and the 1/40 or 1/100 activity against *C*-glycosylated chromone (aloesin) and C8-glycosylated flavones (orientin) in comparison to puerarin (C8-glycosylated isoflavones) (Fig. 2). On the other hand, PurBC was active only on 3″-keto-puerarin, 3″-keto-orientin, and 3′-keto-aloesin. Among them, PurBC showed the highest activity toward 3″-keto-puerarin (Fig. 2).

**Fig 3:**
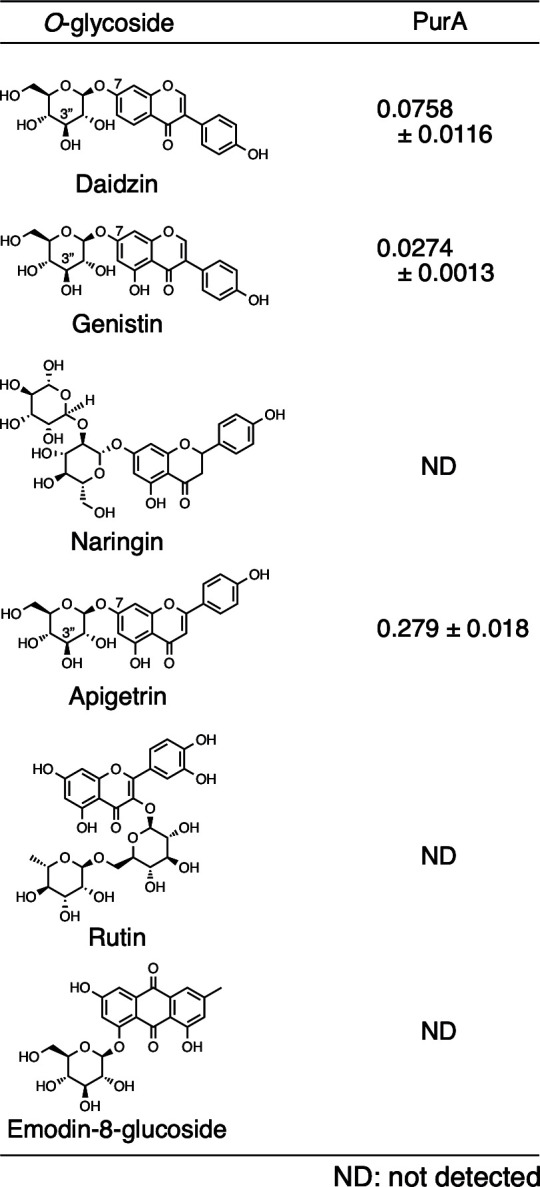
Substrate specificities of PurA and PurBC toward *O*-glycosides.

We also examined *O*-glycosides (daidzin, genistin, naringin, apigetrin, rutin, aloenin, and emodin-8-glucoside) as substrates for PurA (Fig. 3). As a result, daidzin, genistin, and apigetrin were converted by PurA to the corresponding 3′-keto-*O*-glycosides and then subsequently deglycosylated spontaneously to aglycones.

### Database search for PurABC and DgpABC homologs

We investigated the distribution of *C*-glycoside deglycosylation enzymes in nature. PurA and DgpA share no amino acid sequence similarity, whereas PurBC and DgpCB do. Database searches were conducted using PurABC or DgpABC as queries. PurABC homologs were identified in the phyla Actinomycetota, Pseudomonadota, Bacteroidota, Deinococcota, and Arthropoda. In contrast, DgpABC homologs were identified in the phyla Bacillota and Pseudomonadota.

## DISCUSSION

**Fig 4:**
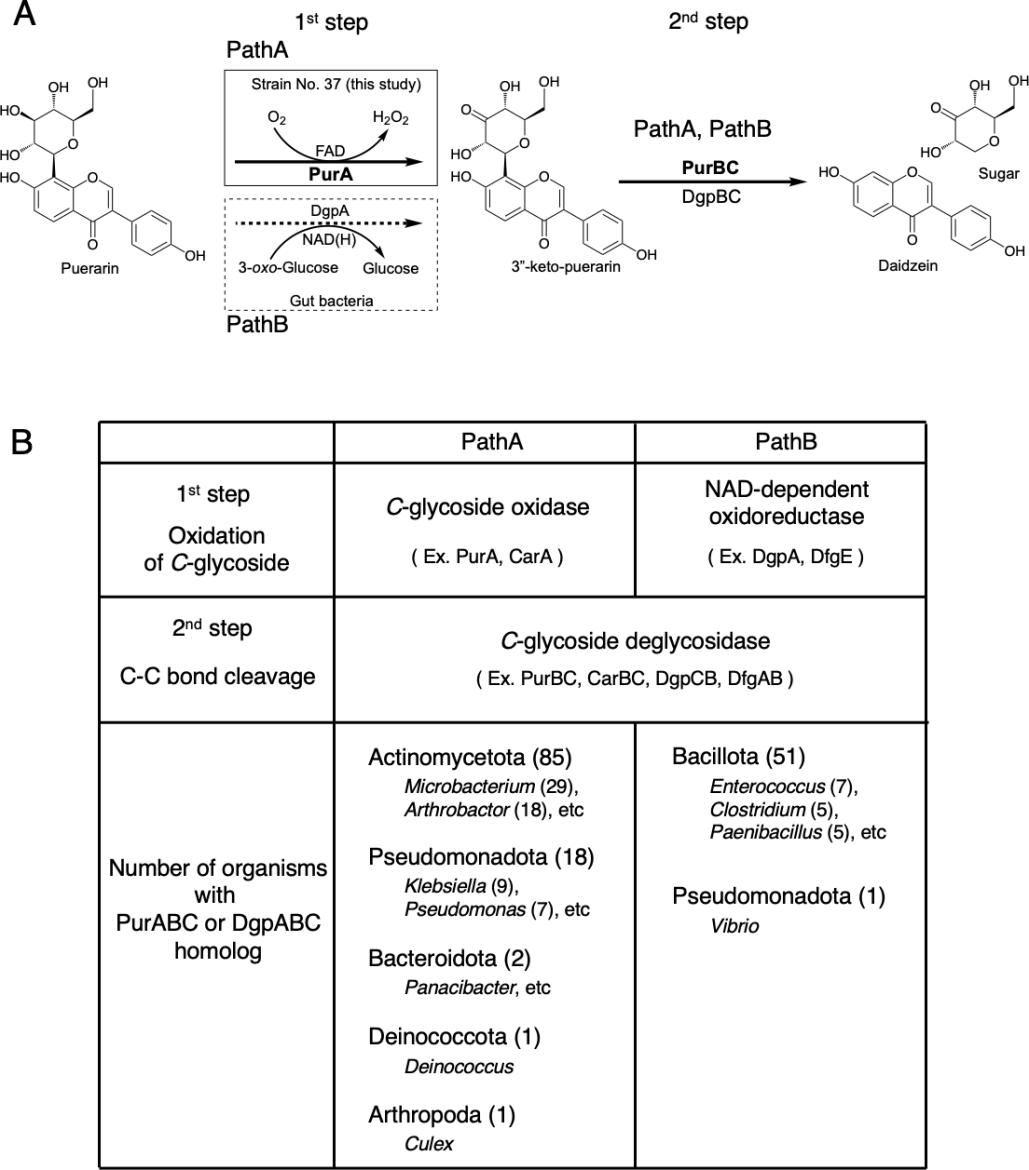
Reaction scheme of PurA, PurBC (PathA) and DgpA, DgpBC (PathB), and the distribution of PurABC or DgpABC homologs in the database. (**A**) Comparison of the puerarin metabolic pathways in strain No. 37 and intestinal bacteria. PurA and DgpA catalyze the same oxidation reaction toward a common substrate and produce the same reaction product; however, they share no amino acid sequence similarity. In contrast, PurBC and DgpBC, which are involved in C-C bond cleavage, exhibit sequence homology with each other. (**B**) The results of the BLAST search using PurABC and DgpABC as queries were selected with an E-value ≤1e-50 and query coverage between 80% and 100%. The results were grouped by phylum, with the numbers in parentheses indicating the number of organisms detected in the BLAST search.

Kakkonto, a Chinese herbal medicine, is utilized as a self-administered treatment for the common cold in its early stages. It is a powder derived from the root of the kudzu plant (*Pueraria lobata*). The root contains C-8 glycosylated isoflavone puerarin (12). Puerarin has been demonstrated to exhibit biological activity, including anti-cancer and anti-diabetic effects (13–17). Following ingestion, puerarin is subjected to deglycosylation by intestinal microorganisms, resulting in the formation of aglycones that are subsequently absorbed into the body and transported to cells, where they exert their biological activities. Accordingly, the deglycosylation mechanism of puerarin by intestinal bacteria represents a subject of considerable interest. The series of studies was reported and finally identified DgpA and DgpBC as deglycosylation enzymes involved in the metabolism of puerarin (6–9, 18) (Fig. 4A). DgpA is an NAD-dependent enzyme that catalyzes the dehydrogenation of C3-OH in the sugar moiety, forming 3″-keto-puerarin. DgpBC is a type of lyase that catalyzes the cleavage of the C-C bond between the sugar moiety and the aglycone (9). Homologs of DgpA and DgpBC have been identified in other intestinal microorganisms (19, 20). This combination for deglycosylation of *C*-glycosides is likely to be conserved among intestinal bacteria. Based on our database search, nearly all organisms harboring DgpABC homologs belonged to the phylum Bacillota, including the genera *Enterococcus*, *Clostridium*, and *Paenibacillus*, whereas only one *Vibrio,* which belongs to the phylum Pseudomonadota, was found to contain a DgpABC homolog (Fig. 4B).

On the other hand, we reported the metabolism of carminic acid, which is the *C*-glycoside used for coloring food, in soil bacteria *Microbacterium* sp. No. 22 and identified metabolic enzymes CarA and CarBC (11, 19). CarA is an FAD-dependent *C*-glycoside oxidase that dehydrogenates C3-OH of the sugar moiety, and CarBC is a lyase that cleaves the *C*-glycosidic bond. While CarBC exhibits amino acid sequence similarity to DgpBC, CarA does not show similarity to DgpA.

It was unclear whether the puerarin degradation pathway of soil bacteria was the same as that of intestinal bacteria or the *C*-glycoside degradation pathway of soil bacteria, because the metabolic fate of puerarin in natural environments has not been reported. In this study, puerarin-catabolizing bacteria were screened, and the most active strain No. 37 was identified as *Paenarthrobacter*. Strain No. 37 represents the first example of a soil bacterium capable of catabolizing *C*-glycosylated flavonoid. This bacterium possesses the ability to deglycosylate puerarin via a two-step enzymatic process, in which both the reaction mechanism and the enzymes involved exhibit similarity to those characterized in the carminic acid metabolism of *Microbacterium* sp. 5-2b, rather than to those found in gut bacteria. The initial reaction involved the oxidation of C3-OH in a sugar moiety, catalyzed by PurA, which resulted in the formation of 3″-keto-puerarin. The subsequent reaction entailed the cleavage of the C-C bond, catalyzed by PurBC, leading to the generation of the aglycone (daidzein) and the sugar moiety (Fig. 4). The amino acid sequences of PurA and PurBC exhibited similarity to those of CarA and CarBC, respectively. Our database analysis revealed that PurABC homologs were mainly present in the phylum Actinomycetota, predominantly soil bacteria (Fig. 4B). In contrast, the microorganisms classified in the phylum Pseudomonadota, Bacteroidota, and Deinococcota also possess PurABC homologs. In comparison, DgpABC homologs were found only in intestinal bacteria, whereas PurABC homologs were present in microorganisms from both natural environments and the human body, such as blood and urine (e.g., *Klebsiella variicola* subsp. variicola and *Klebsiella pneumoniae* [accession numbers CP153984 and CP016811]). Although PurA requires oxygen gas for its reaction, it is distributed more broadly than DgpA.

A comparison of the substrate specificities of PurA and CarA revealed that, while CarA was inactive toward C8-glycosylated compounds, such as puerarin, orientin, and aloesin (11), PurA was capable of utilizing both C6- and C8-glycosylated flavonoids. PurA exhibited relatively broad substrate specificity and represents the first characterized *C*-glycoside oxidase acting on C8-glycosylated flavones and isoflavones. Moreover, PurA showed relatively strong activity toward *O*-glycosides (Fig. 3). Oxidized *O*-glycosides were non-enzymatically deglycosylated as previously reported (11, 21). On the other hand, PurBC, a heterodimer composed of PurB and PurC, exhibited amino acid sequence similarity to *C*-glycoside deglycosylases (CGDs), such as CarBC and DgpBC. Substrate specificity analysis revealed that PurBC, like DgpBC^18^, displayed enzymatic activity exclusively toward C8-glycosylated compounds. Since both 3″-keto-puerarin and 3″-keto-orientin served as active substrates for PurBC and DgpBC, the site of glycosylation appears to be a critical determinant of enzymatic activity rather than the structure of the aglycone (Fig. 2).

In the metal dependency analysis, PurBC exhibited restored enzymatic activity upon the addition of Mn²^+^, Mg²^+^, or Co²^+^ following treatment with chelating agents. In contrast, the enzymatic activities of intestinal bacterial CGDs were recovered by Ni²^+^, Zn²^+^, Cu²^+^, or Fe²^+^, whereas CarBC was reactivated specifically by Mg²^+^. These findings are consistent with the environmental origin of each enzyme, whether it is derived from gut or soil bacteria, suggesting that the growth environment may influence their metal ion preference and enzymatic characteristics.

This study is the first to identify the puerarin-metabolizing microorganism No. 37 (*Paenarthrobacter*) from soil. This strain was isolated by the enrichment culture using the medium that contains puerarin as a sole carbon source. It revealed that strain No. 37 can utilize puerarin as sources of energy and its components. Furthermore, we demonstrated that distinct bacterial species are responsible for puerarin degradation in the natural environment and in the gut, respectively. Notably, the enzyme catalyzing the initial step of puerarin metabolism in soil bacteria differs from the previously characterized counterpart in intestinal bacteria. It was also found that PurABC homologs are distributed across bacteria ranging from those inhabiting natural environments to those associated with humans (Fig. 4). These findings offer new insights into the metabolism of *C*-glycosides in nature.

## Data Availability

Nucleotide sequence data that support the findings of this study have been deposited in the DDBJ/GenBank database under accession numbers LC886353 for *purA*, LC886354 for *purB*, and LC886355 for *purC*.

## SUPPLEMENTAL MATERIAL

Supplemental materials aem.01593-25-s0001.pdf Tables S1 to S5, Figures S1 to S8, and supplemental results.

ASM does not own the copyrights to Supplemental Material that may be linked to, or accessed through, an article. The authors have granted ASM a non-exclusive, world-wide license to publish the Supplemental Material files. Please contact the corresponding author directly for reuse.
