# Supplementary material for: Characterization of the initial degradation mechanism involved in C-glycosylated isoflavone puerarin in soil microorganism
Source: Appl Environ Microbiol. 2025 Nov 20;91(12):e01593-25. doi: 10.1128/aem.01593-25 (PMC12724307; doi:10.1128/aem.01593-25)
Supplement: Supplemental materials — Tables S1 to S5, Figures S1 to S8, and supplemental results. [file aem.01593-25-s0001.pdf]

Supplementally Information

Characterization of the initial degradation mechanism involved in *C*-glycosylated isoflavone puerarin in soil microorganism

Takuto Kumano[1,2,3#]*, Satomi Watanabe[1#], Masakazu Kusakari[1#], Yuzu Terashita[1], Yoshiteru Hashimoto[1,2,3] and Michihiko Kobayashi[1,2,3,4]*

Affiliations

[1] Graduate School of Life and Environmental Sciences, University of Tsukuba, 1-1-1 Tennodai, Tsukuba, Ibaraki 305-8572, Japan

[2] Microbiology Research Center for Sustainability (MiCS), University of Tsukuba, 1-1-1 Tennodai, Tsukuba, Ibaraki 305-8572, Japan

[3] Tsukuba Institute for Advanced Research (TIAR), University of Tsukuba, 1-1-1 Tennodai, Tsukuba, Ibaraki, 305-8577, Japan

[4] Center for Quantum and Information Life Science (QILS), University of Tsukuba, 1-1-1 Tennodai, Tsukuba, Ibaraki 305-8572, Japan

# These authors contributed equally to this work.

Co-corresponding authors:

Michihiko Kobayashi, Ph.D. (Professor)

Tel: +81-29-853-4628, Fax: +81-29-853-7299

E-mail: kobayashi.m.fe@u.tsukuba.ac.jp

Takuto Kumano, Ph.D.

Tel: +81-29-853-8692, Fax: +81-29-853-7299

E-mail: kumano.takuto.fu@u.tsukuba.ac.jp

Supplementary Results

Nucleotide sequence of *purA*

1-ATGGACATTGAAACAGCATCAATCCGCCCTTGGGATCTCGTCATCATTGGCAGCGGTCCG
61-AACGGCGCTGCAATCGCCCATGAAGTTCATCGGGCCTCGCCGGAGGCATTCATCCTGGTC
121-CTCGAGAGTGGCCGAGGAATGAGCACCAACCAAGGCGAACACCTTGTAGAGTCCGACGAG
181-GGGGCCCTGCGCGAAGCGTTCGAGACGCTGATGCGTCGCGCCCGGCAGATCGAGTACGTC
241-AAGAACGCCGGAGCCGTACTCGGCAGTGACGATGGCTGGACCCCAGAAGGAACCGGCATT
301-TTCCCTGTCTCCTACCTCGGCCACGATTTCACTGAGTTCTCCGGGGCTTCCTTTTCGTGG
361-AATGTCGGGGGAATGGGGATTCACTGGGCGGCAGCGTCACCCTCGCCATACGCCGATGAG
421-ATCCCGGGGTTCGCAGGCAGGGACTTCGCTGACGACCTTCAAACGGCGAAACGGTTGCTC
481-CGCGTACACCCACAGGCGTTCACCGGCAATCCATATCGAGAACCAATCCTCACCGCACTC
541-CGAGCCGCGGTCCCTTCGGACATCCACGGCCGAGAGGCCCAGGACATGCCGCTCGCAGGT
601-CTGCGCCGGACCGGTGGGGGCTCATTCGCCCGAACCGGTCCCCGTGACATCGCGCCCGAA
661-CTCTTCGACGAATCCGCTCCGCACATCGCACTGGTCTCAGGTACTCTTGCGACCCGGATC
721-CTGCACAGCAACGGAAAGGTCAGCGCGGTGGTGGCACGTGACCTGGCCACTGGTACCGAG
781-CGCGAAATACCCGCGAGGTCAGTGGTCGTCGCCGCAGACACCTTGCGCAGCCCGCAGATC
841-CTGTGGGCCTCGAAAATCCGGCCCCACGCGTTAGGGCTCTACCTGAACGAACACGTTTCC
901-ATCGACGGAAGCGTAATTGTCGATAGGCCAAAGCTCGGCTTATCCGAAACCAAGCTACCC
961-AAGCCGGAGCTGAATGAACCGTTCGTCGGAGCCTACTGGAGTCCCTCCATCGGCGCCGAG
1021-CGGCCCACACACGGGCAGATGATGGAAACCTTCGATGGCCGTGGGCACCGTATCGGCATG
1081-AGCTGGTACACGAATACGGACCTCCGTGCAGAGAACCGTATCGAATTCTCCGACGCAGAG
1141-ACGGACGAATTGGGAATGCCACTCATGAAGGCTCACTTCGCGTACACGGACGCGGACCGG
1201-AGGAGGATCGAAATGCTCCGCGATGTGCAGCGCCGCGCAGCCACCGCCATCGGGGAGTTC
1261-CTGCCTGGCGACTCCGAGACACTCGCACCCGGATCATCGCTGCACTACACGGGAACGGTG
1321-CGTCTGGGATCCACCGATGACGGAAAGAGCGTCGCGAGCCCGGACGGACTGGTCTGGGGC
1381-TTCGACAATCTCTTCGTGGCTGGAAACGGTGCTGTGCCCACTGCGCTGACCTGCAACTCG
1441-ACGCTGACCGGAATGACGCTGAGCGTGAGGACCGCCCGGGCGGTTACCCGCCACATTTCC
1501-AGCGAAAGTGCCCTTCCGCGGGTTATGCGCGGAGCGGGAACGGGTTACTGA (1551 bp)

Amino acid sequence of PurA

1-MDIETASIRPWDLVIIGSGPNGAAIAHEVHRASPEAFILVLESGRGMSTNQGEHLVESDE
61-GALREAFETLMRRARQIEYVKNAGAVLGSDDGWTPEGTGIFPVSYLGHDFTEFSGASFSW
121-NVGGMGIHWAAASPSPYADEIPGFAGRDFADDLQTAKRLLRVHPQAFTGNPYREPILTAL
181-RAAVPSDIHGREAQDMPLAGLRRTGGGSFARTGPRDIAPELFDESAPHIALVSGTLATRI
241-LHSNGKVSAVVARDLATGTEREIPARSVVVAADTLRSPQILWASKIRPHALGLYLNEHVS

301-IDGSVIVDRPKLGLSETKLPKPELNEPFVGAYWSPSIGAERPTHGQMMETFDGRGHRIGM
361-SWYTNTDLRAENRIEFSDAETDELGMPLMKAHFAYTDADRRRIEMLRDVQRRAATAIGEF
421-LPGDSETLAPGSSLHYTGTVRLGSTDDGKSVASPDGLVWGFDNLFVAGNGAVPTALTCNS
481-TLTGMTLSVRTARAVTRHISSESALPRVMRGAGTGY (516 *aa*)

Nucleotide sequence of *purB*

1-ATGACCGCATCGAACAACTACTCGTTGCCAACAATTGGGTCCGGCCTGACCCTATATTCC
60-TTCACGAATGAGTGGCTCAGCGGGCAATTCGACCTGGAGCAAATTCTGCGCGAAGTTGCC
121-GCACGGGGACTGGGCCCGGGGGTGGAGGTCGTCGGGTATCAGTCACTGCGAAGCTTCCCC
181-GACATCGACGATGCGACGACAGACAAGTGGCATGGCCTCATCGACGAGCTTGGCTTGGTT
241-CCCACTTGCCTGAGCTCAAATGTGGACATTGCGCTTCGCTCAGATCGGTTCCTGAATGCC
301-GATGAGATGACAGAGCTGCTCGAACGGCAACTTCGCACAGCAAATAAGCTCGGGTTCAAC
361-ATCGTACGTATCCAAATCGGGGCGTCGGCAGAAGTAATCCAGCGGGTCACTCCGCTTGCT
421-GAGGACCTGGGCCTGCGCATGGGTATGGAACTCCACGCACCGGAGGGACCACGCACCGAG
481-AGCATCATGCGTGTGCGCGACCTCTACGCCGAACTTGATTCGCCGGCACTGGGATTCATC
541-CCCGACTTCAGCGCCACAATGCGTGACATACCCCTGACGGAGCAGCACCAATGGGTCGAA
601-GCCGGCCTGCCCCAGGATCTCGTGGACATTTTCGTTGAGAACTGGCGAACCGCGCCCGGC
661-ACAATCAAAGACCGTTTTCAGGCATTCCGCAGGCTGGCACTCGCGCGCGGCGCCTCGGAA
721-GAAGCAATCGCTCCGACGGTAGGTGCGCTGACAATGCACGGCACAGAGCCAATGGAGAGC
781-TGGTATGACATCGCCGACCAGATCATCCATGTCCACGGCAAGTGCTACGAGTTTGATGCC
841-GTCGGTGACGAGCCCAGCATCGACTATGACGCTGTCGCTCGCCTGCTGGTCGATATTAAC
901-TACAAGGGCTATATCTCCACCGAATGGGAGGGGCACTACTTCGCCTCCTCCAACGTCAGC
961-GCTTTCGACCAGGTGCAGGCCCACCAGAGACTATTGGGCCGCAGCTTGGAAAAGGCAGCC
1021-GGTACGCGCATCTAA (1035 bp)

Amino acid sequence of PurB

1-MTASNNYSLPTIGSGLTLYSFTNEWLSGQFDLEQILREVAARGLGPGVEVVGYQSLRSFP
61-DIDDATTDKWHGLIDELGLVPTCLSSNVDIALRSDRFLNADEMTELLERQLRTANKLGFN
121-IVRIQIGASAEVIQRVTPLAEDLGLRMGMELHAPEGPRTESIMRVRDLYAELDSPALGFI
181-PDFSATMRDIPLTEQHQWVEAGLPQDLVDIFVENWRTAPGTIKDRFQAFRRLALARGASE
241-EAIAPTVGALTMHGTEPMESWYDIADQIIHVHGKCYEFDAVGDEPSIDYDAVARLLVDIN
301-YKGYISTEWEGHYFASSNVSAFDQVQAHQRLLGRSLEKAAGTRI (344 *aa*)

Nucleotide sequence of *purC*

1-GTGAGTGCCACTGAAAGAGTGATTGGTGGGGACGAAGTCCGGGTCAACCAGGCGGGGAAT
61-GCGCAAGTTACCTTGCGCCTGCCTTGGTACCGGAGCCTCGCTCCGTCAACTGTTGAAGAC

121-ATCGCCGTCTCGATTGACGGCCACACCATACCTCGAAATGAACTGACTGTAGAGATCAAC
181-GGGATCGAAAGCGAGCTCGACGCCATCGCGGATCGGTGGCAGGAGACCTGGTTCGTCCAG
241-GACCGGGCCATCGTTCGCTTCCCGACCCCAGGAGATCTCAATGGTGCCGTCAATACGACG
301-CTATCCATCACGCTCCGGATCCCATACATCCTAACCGGCCCTGATTCCGCGCTGAAGCGG
361-AGCACCAGTGAGACCCGCAAGCTTTCTGTCGTCCGTGAGGAGCAGCCATGA (411 bp)

Amino acid sequence of PurC

1-VSATERVIGGDEVRVNQAGNAQVTLRLPWYRSLAPSTVEDIAVSIDGHTIPRNELTVEIN
61-GIESELDAIADRWQETWFVQDRAIVRFPTPGDLNGAVNTTLSITLRIPYILTGPDSALKR
121-STSETRKLSVVREEQP (136 *aa*)

Supplementary Tables

Table S1 Bacterial strains and plasmids used in this study.

**Strains and plasmids**	**Relevant characteristics**
**Strains**	
E. coli DH10B	Cloning host; F^-, *mcrA*, Δ(*mrr-hsdRMS-mcrBC*), ϕ80d*lacZ*ΔM15, Δ*lacX74*, *recA1*, *deoR*, *endA1*, *araD139*, Δ(*ara, leu*)7697, *galU*, *galK*, λ^-, *rpsL*, *nupG*
E. coli BL21 (DE3) RIL	
E. coli Rosetta 2 (DE3)	Strain for recombinant protein expression; F^-, *ompT*, *hsdS*$_B$(r_B^- m_B^-), *dcm* (DE3), pRARE2 (Cam^R)
Paenarthrobacter sp. No.37	Wild-type puerarin-catabolizing strain
**Plasmids**	
pET24a(+)	T7 RNA polymerase-dependent recombinant protein expression vector, Kan^R.
pET24a(+)-His-*purA*	The His-tag added *carA* fragment (1551 bp) was inserted into the *Nde*I and *Eco*RI sites of pET24a(+)
pET24a(+)-*purBC*	The *purBC* fragment (1506 bp) was inserted into the *Nde*I and *Eco*RI sites of pET24a(+)

Table S2 Primers used in this study.

**Primers used for cloning of *purA* and *purBC***	
purA- Fw (*Nde*I)	TAAGAAGGAGATATACATATGGACATTGAAACAGCATCAATCCGCCCTTGGG
purA-C-His Rv (*Hin*dIII)	CTCGAGTGCGGCCGCAAGCTTGTAACCCGTTCCCGCTCCGCG
purBC Fw (*Nde*I)	TAAGAAGGAGATATACATATGAGTGCCACTGAAAGAGTGATTGGTGGG
purBC Rv (*Eco*RI)	TTGTCGACGGAGCTCGAATTCTTAGATGCGCGTACCGGCTGCC

The restriction site is underlined.

Table S3 Purification table of PurBC.

Step	Total protein (mg)	Total activity (μmol/min)	Specific activity (μmol/min/mg)	Yield (%)	Purification (fold)
$(NH_4)_2SO_4$	1.59	15.6	0.133	100	1
Butyl 650M	0.92	11.2	0.290	71.8	2.18
Resource Q	1.15	1.49	0.323	9.5	2.44
Bioassist Q	0.34	0.505	0.990	3.2	7.47
Superose 12	0.08	0.206	2.53	1.3	19.1

Table S4 Effects of metals on the activities of PurA and PurBC.

Metals	Relative activity (%)	
	PurA	PurB
Control	100	100
LiCl	146±10	95.6±1.1
NaCl	134±6	92.7±0.8
$MgCl_2$	78.2±5.8	137±2
$CaCl_2$	82.2±3.7	68.2±10.4
$BaCl_2$	147±5	88.5±2.7
$MnCl_2$	2.82±0.38	192±0.4
$ZnCl_2$	1.61±0.01	ND
$CdCl_2$	22.1±8.3	32.5±0.6
$CoCl_2$	8.36±0.88	114±2
$AlCl_3$	65.4±3.4	73.2±1.2
$PbCl_2$	0.322±0.068	2.11±0.10
$HgCl_2$	19.3±1.7	ND
$NiCl_2$	108±1	31.8±0.4
$CuCl_2$	13.9±6.2	ND
$FeSO_4$	ND	64.2±4.1
$FeCl_3$	ND	121±3
$RbCl_2$	106±1	105±3
$SrCl_2$	120±2	89.7±2.4
CsCl	102±4	101±0
$NaMoO_4$	87.7±2.4	102±1

ND: not detected

Table S5 Effects of small-molecular compounds on the activities of PurA and PurB.

Compounds	Relative activity (%)	
	PurA	PurBC
Control	100	100
**SH inhibitors**		
5,5’-Dithio-bis-2-nitrobenzoate (DTNB)	105±7	125±3
Iodoacetate	90.3±9.5	121±1
N-Ethylmaleimide (NEM)	101±4	123±7
p-Chloromercuribenzoate (PCMB)	73.0±5.9	7.34±2.96
**Carbonyl reagents**		
Hydroxylamine	49.1±0.4	124±4
Phenylhydrazine	73.2±4.5	89.6±5.4
Semicarbazide	84.5±4.4	108±4
Aminoguanidine	89.6±2.2	121±2
**Chelators**		
α,α'-Dipyridyl	78.7±2.2	50.8±3.2
o-Phenanthroline	ND	10.1±0.2
Ethylenediaminetetraacetic acid (EDTA)	134±4	89.0±1.5
Diethyldithiocarbamate	132±4	45.4±4.7
NaN_3	131±3	102±1
KCN	139±2	47.3±1.4
**Reductants**		
Dithiothreitol (DTT)	106±2	33.8±0.3
2-Mercaptoethanol	132±1	107±2
$Na_2S_2O_4$	142±3	101±1
**Oxidants**		
H_2O_2	140±4	114±3
Ammonium persulfate	45.5±0.3	110±2
**Serine modifier**		
Diisopropyl fluorophosphate	38.1±0.1	85.0±1.4

ND: not detected

Supplementary Figures

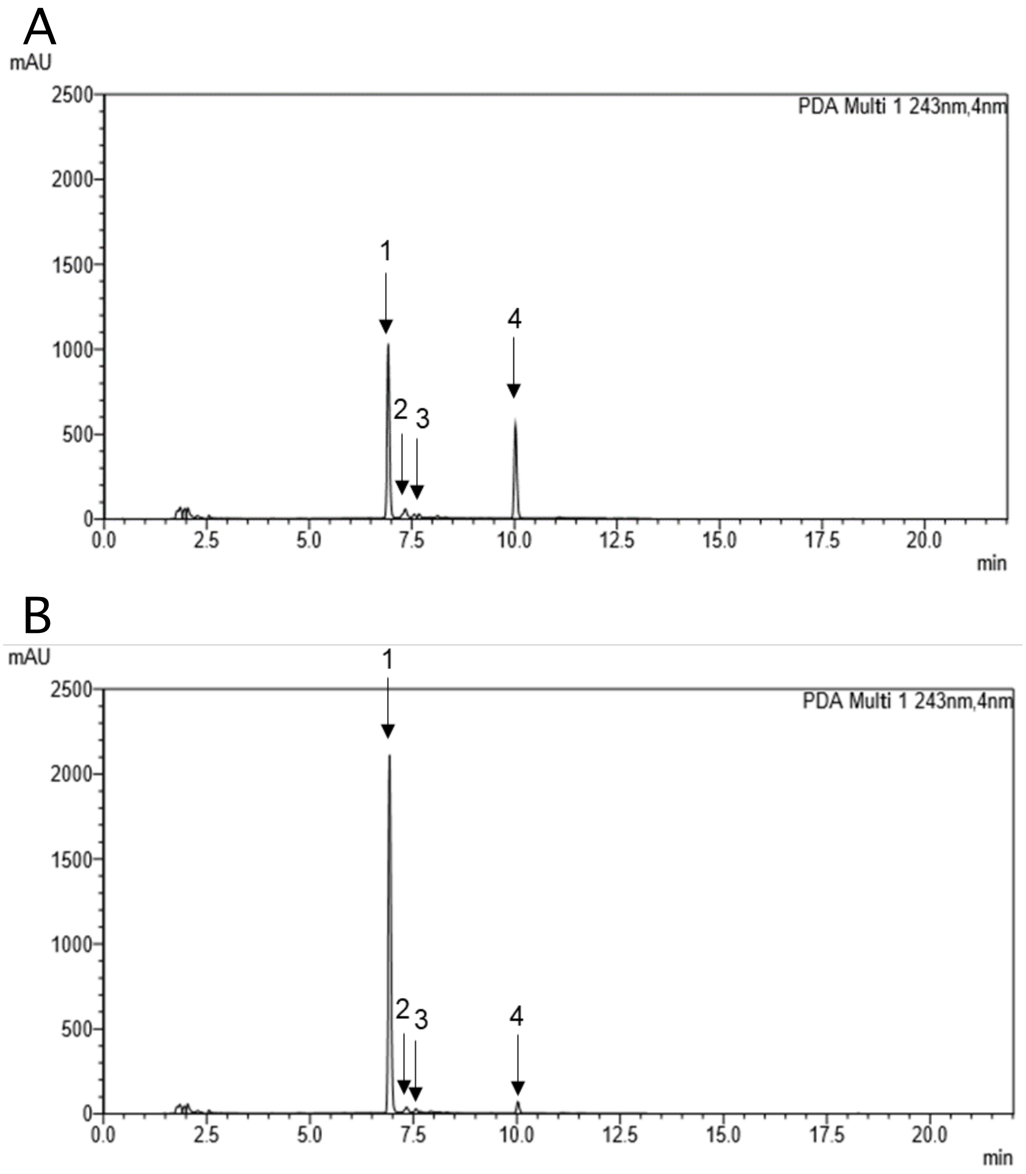

Fig. S1 Induction of puerarin degradation activity.

High-performance liquid chromatography (HPLC) chromatogram of puerarin degradation assay using cell-free extracts (CFE) of No.37. Puerarin was added (**A**), and was not added (**B**) to the medium. The following compounds were identified: **1** puerarin; **2** and **3** 3''-keto-puerarin and its isomer; **4** daidzein.

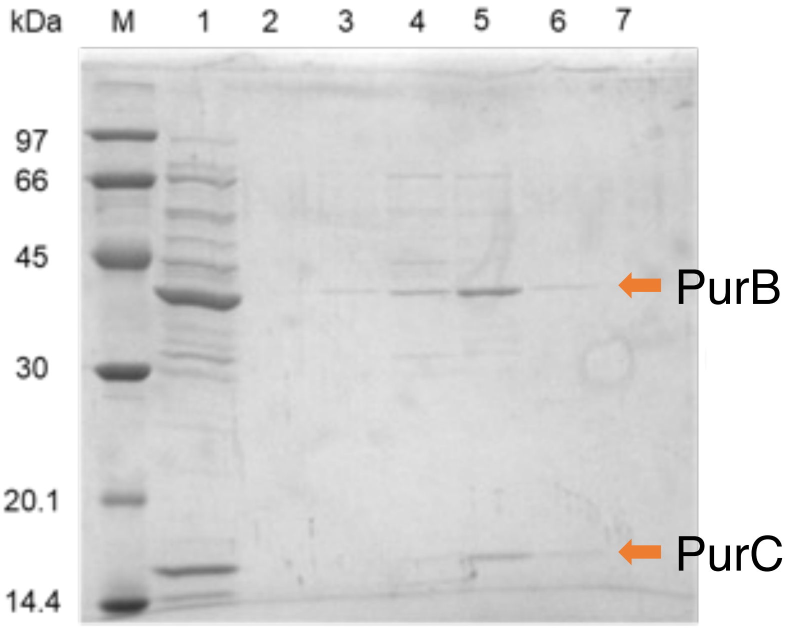

Fig. S2　Purified PurBC from *Paenarthrobacter* sp. No. 37.

This SDS-PAGE figure shows proteins purified by using Superose 12 column.　Lane M, LMW marker; Lane 1, sample prior to column chromatography; Lane 2-7, fractions resulting from column chromatography.　The size of PurB and PurC is indicated by orange arrows.

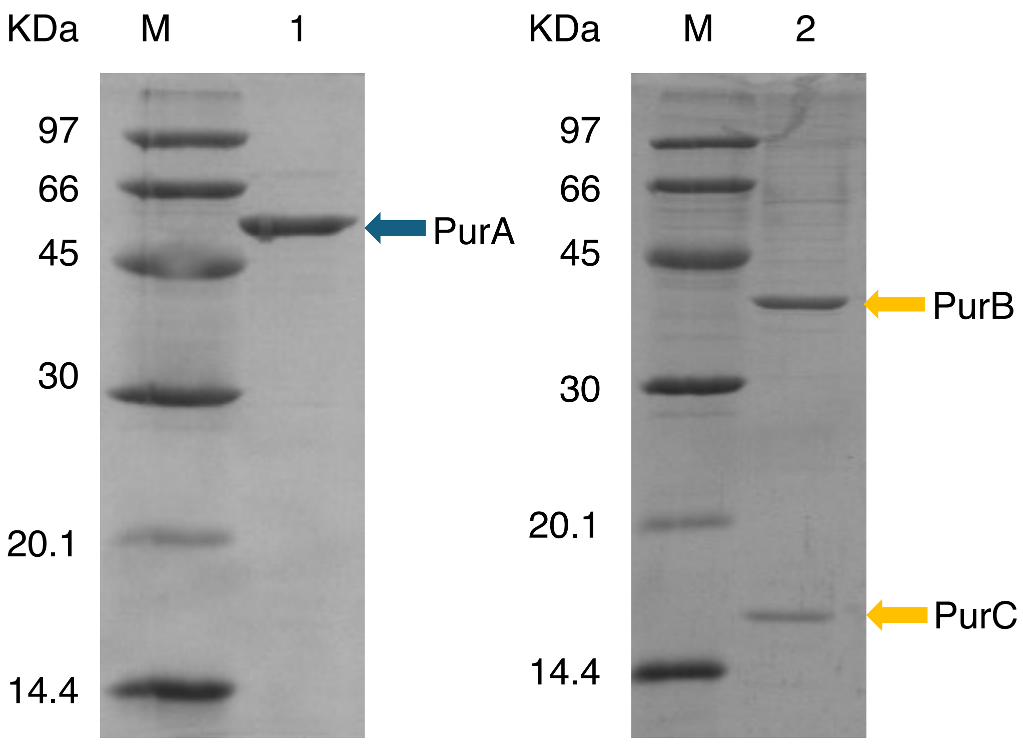

Fig. S3 Recombinant PurA and PurBC expressed in *E. coli*.

SDS-PAGE of heterologously expressed proteins purified by Ni-NTA chromatography. Left: PurA; Right: PurBC. Blue and orange arrows indicate PurA, PurB, and PurC, respectively.

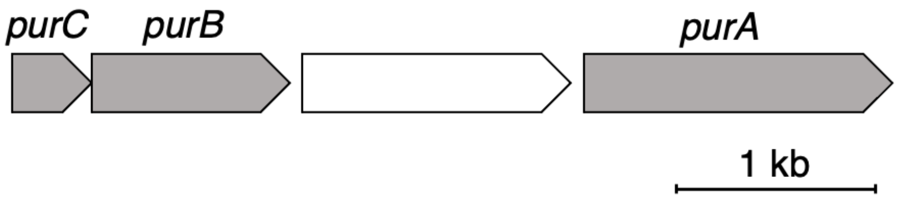

Fig. S4 Gene cluster containing *purA*, *purB* and *purC* in the genome of strain No. 37.

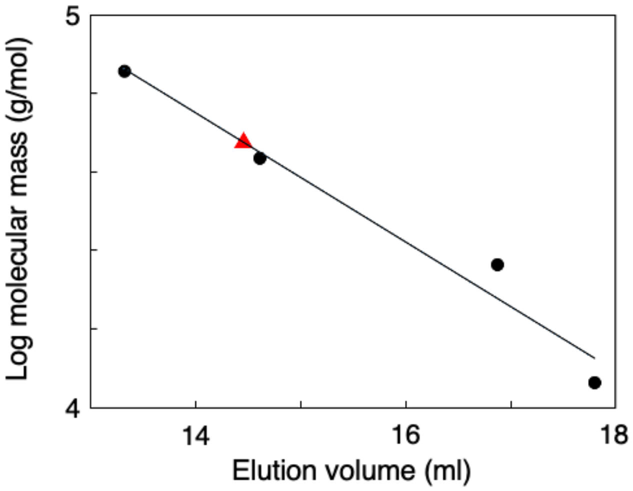

Fig. S5　Size-exclusion chromatography of PurA.

Molecular weight of protein marker was 67, 43, 25, 12.4 kDa.　A red triangle represents PurA.

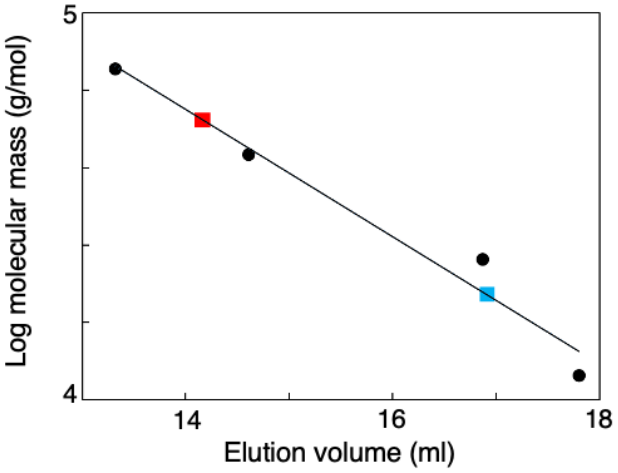

Fig. S6　Size-exclusion chromatography of PurBC.

Molecular weight of protein marker was 67, 43, 25, 12.4 kDa.　A red square represents PurBC and blue square represents PurB.

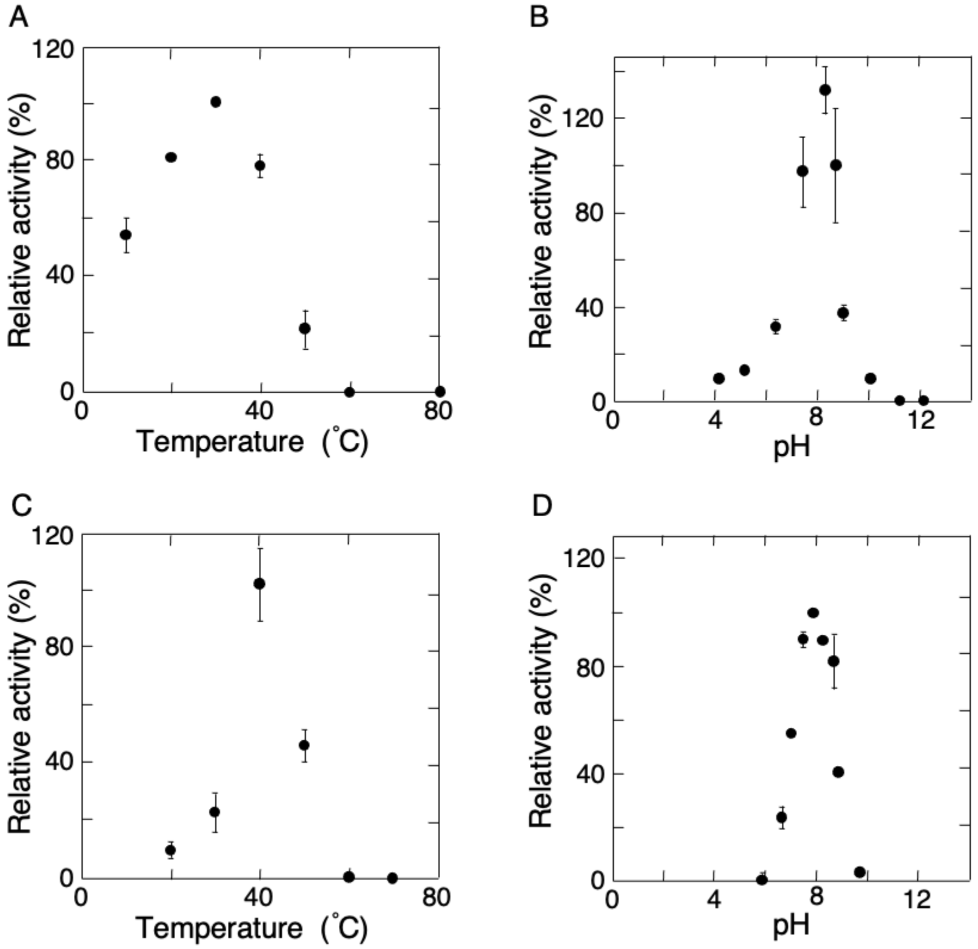

Fig. S7 Effect of temperature and pH.

A: relative activity of PurA in various temperature. B: relative activity of PurA in various pH.

C: relative activity of PurBC in various temperature. D: relative activity of PurBC in various pH.

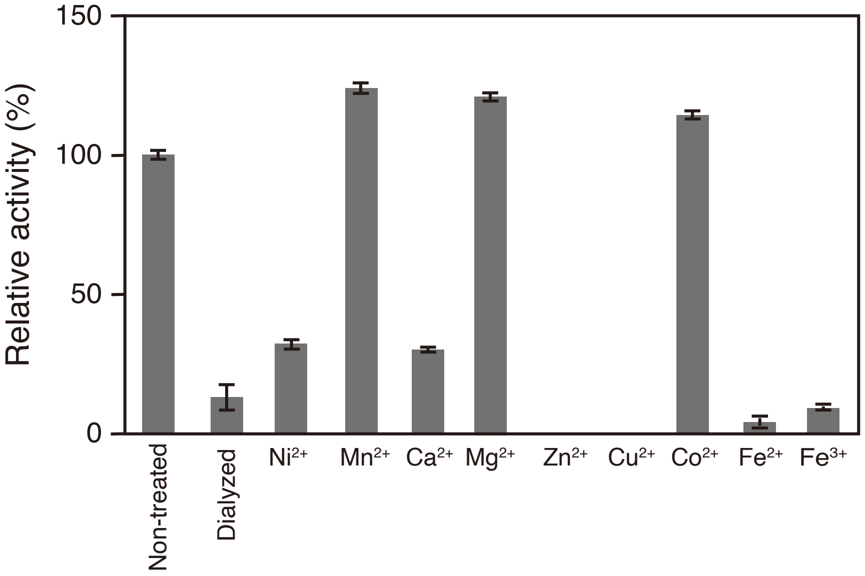

Fig. S8 Metal ion dependency of PurBC.
